# Analysis of the Material Removal Process in Precision Milling of AZ91D Magnesium Alloy

**DOI:** 10.3390/mi16111283

**Published:** 2025-11-13

**Authors:** Jarosław Korpysa

**Affiliations:** Department of Production Engineering, Lublin University of Technology, 20-618 Lublin, Poland; j.korpysa@pollub.pl

**Keywords:** precision milling, magnesium alloy, chip formation, burrs, ploughing

## Abstract

The study investigated the material removal process during precision milling of AZ91D magnesium alloy. A high-speed camera enabling high-frequency image recording was used to observe the cutting zone. In effect, it was possible to observe the mechanism of the chip formation process at different stages of the cutting flutes performance. Experiments were conducted with different feeds per tooth in order to detect the occurrence of ploughing. Results showed that the both cutting flutes of the end mill did not perform in a uniform manner. Material was predominantly removed by first flute, as a result of which chips formed by this flute were much larger than those generated by the other flute. Nevertheless, the shearing process proceeded effectively even at low feed values. Results also showed that large burrs were formed when machining was conducted with low feed per tooth, which confirmed a significant contribution of plastic deformation to burrs formation. An increase in feed per tooth, however, made it possible to minimize the phenomenon of burrs formation.

## 1. Introduction

Magnesium alloys are known for their very low density and good strength-to-weight ratio. This makes them an attractive material for manufacturing components requiring the lowest possible weight. As a result, they are increasingly used in the aerospace, automotive, or military industries. However, despite these advantages they are also prone to spontaneous combustion, which poses significant difficulties in the context of machining. Magnesium dust in particular is susceptible to ignition and explosions, which is why abrasive machining of magnesium alloys is not carried out due to very large quantities of generated dust [1,2]. This means that the high quality of manufactured parts must be achieved using other machining methods. Precision milling appears to be an alternative method for increasing safety while simultaneously improving quality. Precision milling is a variant of machining in which very small values of undeformed chip thickness are removed. However, using excessively small undeformed chip thickness values lead to a phenomenon known as material ploughing. In such cases, the material is not effectively removed but instead undergoes plastic deformation. This has a detrimental effect on the machining process, as plastic deformation significantly deteriorates the condition of the machined surfaces [3,4,5]. Additionally, material deformation contributes to the formation of burrs along the edges of the machined features. These burrs also negatively affect surface quality and must be removed in a subsequent process, most often through a brushing [6] or grinding operation [7]. This necessitates additional operations, which extend the overall machining time and consequently increase the production costs. It is therefore essential to ensure the proper execution of the material removal process, which guarantees the achievement of the required machining outcomes. To make this possible, monitoring and control of the machining process are necessary [8,9]. For process observation, cameras and vision systems can be utilized.

High-speed cameras are a key tool in modern industry and scientific applications. They have a unique capability of capturing images at a rate of several thousand or even millions of frames per second, which makes it possible to capture phenomena that are invisible to the human eye or standard industrial cameras. As a result, one of the main areas of application for high-speed cameras is the monitoring and analysis of machining processes. The high operating frequency of such cameras allows them to accurately capture rapidly varying phenomena, which makes them ideal for use in studies on machining. They allow accurate analysis of the cutting tool–workpiece contact and thus enable a better understanding of machining process mechanisms. High-speed cameras can also be integrated into real-time visual inspection systems, which allows for rapid detection of anomalies and changes in machining conditions [10,11].

Studies have also shown the possibility of using high-speed cameras for precise measurements of cutting tool geometry, in order to enhance machining accuracy. As a result, it is possible to accurately determine their technical condition and detect even small changes in cutting edge geometry [12,13]. In addition, high-speed cameras can be used to automatically detect cracks in and wear of cutting tools, which allows for rapid response and tool replacement. The use of such cameras also makes it possible to detect tool defects, which is especially important when it comes to thin-walled parts [14]. The integration of high-speed cameras with machine tool control systems enables automatic positioning of cutting tools [15,16,17] and workpieces [18,19]. Visual inspection systems analyze the position of the cutting tool relative to the workpiece, which makes it possible to eliminate positioning errors and increase machining precision. Automatic tool position correction can significantly affect the quality of manufactured parts and reduce material losses. With advanced imaging techniques, it is possible to detect dimensional and geometrical deviations as well as surface deformations [20,21,22]. Data obtained from such images can then be used to correct the machining strategy and thus enhance the accuracy of workpieces. The control of manufacturing errors is crucial to ensure the required product quality, especially in precision machining. Properly configured visual inspection systems enable accurate surface mapping and comparison of obtained results with the model, which allows detection of even the smallest deviations [23]. To ensure the required quality is reached, it is also important to use machines with both dynamic and thermal stability [24,25]. Despite their numerous capabilities, high-speed cameras are still most often used for cutting process analysis and chip formation observation [26,27]. Based on images captured by high-speed cameras, one can also optimize parameters of the cutting process. A visual analysis makes it possible to tailor machining conditions to characteristics of the workpiece material, thus enhancing machining efficiency or reducing tool wear.

Denkena et al. [28] employed a high-speed camera to investigate the orthogonal cutting of hybrid components made of SAE1020 and SAE5140. The study focused on variable cutting speed, uncut chip thickness and cutting edge rounding. Results demonstrated that these parameters had a significant impact on chip thickness and shear angle. Machining conditions were also found to affect surfaces and cutting forces. The use of a too small uncut chip thickness changed chips from continuous to segmented. Schneider et al. [29] also conducted orthogonal cutting on pure titanium grade 2. A scanning electron microscope was used for observations. The variables were undeformed chip thickness and geometry of the cutting edge. When the cutting process was conducted with the undeformed chip thicknesses of 5.2 µm and 10 µm, segmented chips formed regardless of the cutting edge geometry. However, decreasing the undeformed chip thickness to 0.4 µm led to the formation of continuous chips only when a sharp edge was used. The use of a tool with a sharp edge resulted in the formation of thicker chips and had a positive effect on cutting forces and surface texture. Denkena et al. [30] investigated the impact of metal working fluids on chip formation in orthogonal cutting of AISI4140 steel. Results demonstrated that the use of cooling led to reduced tool–chip contact length and to reduced chip curl. A further change in this respect was induced by increasing the cooling pressure from 10 bars to 30 bars. This relationship was observed for the tool with a polished and ground rake face. Wet cutting also led to a decrease in cutting force. Liu et al. [31] focused on the orthogonal cutting of potassium dihydrogen phosphate (KDP). The process of chip formation was investigated over a wide range of undeformed cutting depth from 0.1 to 16 µm. Depending on the cutting depth, three cutting modes and three types of chips were identified. The use of a low uncut chip thickness value resulted in a very small built-up edge. An increase in uncut chip thickness led to the formation of continuous chips first and then to chip curl. Molnar et al. [32] investigated the mechanism of chip formation in orthogonal cutting of 2024-T351 aluminum alloy. The use of uncut chip thickness of 100 µm led to the formation of continuous chips, while with an uncut chip thickness of 400 µm one could observe chip segmentation. The presence of chip segmentation was also due to the use of a low cutting speed and small rake angle. An increase in the cutting speed caused an increase in the quantity of segmented chips. Captured images also helped determine a shear angle of approx. 30–40°. Berezvai et al. [33] showed the potential of using captured images for dynamic phenomenon analysis. An algorithm was developed for chatter detection based on images. Cotterell et al. [34] used a high-speed camera to investigate the orthogonal cutting of Ti-6Al-4V titanium alloy. Captured images were used to determine chip thickness and chip–tool contact length. Results were used to calculate shear strains, showing that they increased with cutting speed. The impact of variable feed rate on shear strain was small. Harzallah et al. [35] investigated the orthogonal cutting of Ti-6Al-4V, with focus put on in situ measurements of kinematic fields. A high-speed camera was used to observe a cutting process conducted with a cutting speed of 3 m/min and 15 m/min. It was shown that the use of a higher cutting speed induced pure shear of material and reduced the primary shear zone. An algorithm was developed for calculating strain and strain rate based on captured image analysis. The use of a higher cutting speed led to a slightly lower strain while the strain rate was several-fold higher. Che et al. [36] employed a high-speed camera to observe chip formation in orthogonal cutting of C45E4 steel. The study demonstrated that the chip formation process proceeded gradually and that chip curl occurred when chips reached a certain length. They were described as jagged chips. The cutting process was also simulated, and the numerical results showed high agreement with experimental findings. Wang et al. [37] focused on the problem of material removal in orthogonal cutting of cortical bone samples. Experiments were conducted with a high-speed microscope. It was observed that serrated chips were formed whatever the cutting depth. The use of higher cutting speeds led to a decrease in segment width, despite no variation in the shear plane. This resulted in reduced energy consumption. Davis et al. [38] used a high-speed camera to investigate a turning process for magnesium-based metal matrix composites. The use of imaging made it possible to observe changes in formed chips with varying machining parameters. An increase in cutting speed changed the chip shape from saw-tooth to particle chips. The impact of other machining conditions on chip shape was practically insignificant. The orthogonal turning of grade 4 titanium was investigated in [39]. It was shown that a low cutting speed led to a cyclic share of chip formation. In contrast, the use of a higher cutting speed led to a uniform share of chips and increased spacing between shear bands. High-speed camera-captured images can also serve for chip measurements. Moraiti et al. [40] presented that an increase in cutting speed primarily resulted in higher shear angle and reduced compression ratio. An increase in uncut chip thickness had an adverse impact on the two parameters, as it led to a reduced compression ratio and, at the same time, increased shear angle. Several studies also investigated the use of high-speed cameras to observe milling processes. Li et al. [41] used such a camera to investigate the chip flow direction in up and down milling of Ti–6Al–4 V titanium alloy. It was demonstrated that regardless of the machining type, chips were susceptible to adhering to the machined surface. The quantity of adhered chips was nonetheless smaller in down milling, which resulted in better surface quality. Fecova et al. [42] also investigated up and down milling processes, but with respect to C45 steel. The study focused on chip formation. The use of a high-speed camera allowed only general observations due to limited access to the cutting zone. Nonetheless, the results showed that the shape of formed chips differed depending on the machining direction. Azmi et al. [43] also used a high-speed camera for chip observation during the milling of glass fiber-reinforced polymer composites (GFRP). As a result, different mechanisms of chip formation were identified, their nature being more complicated than for standard materials. It was shown that chip formation was successful when machining was performed in the fiber direction, while the 90° angle milling would produce dust and practically no chips. Results also showed the presence of delamination.

Despite the growing interest in precision machining and micro-milling, the number of papers on magnesium alloys that has been published to date is very small. Bhagat et al. [44] performed micro-milling of the AZ31B magnesium alloy. The authors analyzed cutting force and surface roughness depending on the change in spindle speed and feed. Increasing the spindle speed caused a decrease in cutting force and an improvement in the surface. It was also shown that the size effect was observed mainly for *f_z_*/*r_n_* below 0.5. In this range, there was also a significant increase in specific cutting energy and broken chips dominated. Ercetin et al. [45] performed micro-milling of TZ54 magnesium alloy. In the case of this material, cutting force decreased due to increasing cutting speed and increased with increasing depth of cut. A clear deterioration of surface quality, large burr width and increased cutting force indicated that the minimum chip thickness was about 7–34% of *f_z_*/*r_n_*. Similar studies [46] were conducted on Mg13Sn magnesium alloy. Increasing feed and depth of cut resulted in increased cutting force and surface roughness. Increasing cutting speed had the opposite effect, as it caused a decrease in the analyzed parameters. The occurrence of ploughing signs on the workpiece surface was confirmed when using the lowest feed. Suneesh et al. [47] focused on the optimization of micro-milling of Mg-3.0Zn-0.7Zr-1.0Cu magnesium alloy. The studies showed that the feed had the greatest influence on the machining, and its increase resulted in the improvement of the surface quality while increasing the cutting forces and tool wear. The deterioration of these parameters also occurred due to the increase in the spindle speed. The optimal solution was to use the lowest values of cutting parameters—*n* = 8000 rpm, *f_z_* = 0.5 µm/tooth, and *a_p_* = 30 µm.

A review of previous studies on the use of high-speed cameras and visual inspection systems for analyzing material cutting processes demonstrates that most of these studies focused on turning and orthogonal cutting processes. This primarily results from their kinematics. With these processes, it is possible to set the camera in such a way to enable an ongoing observation of material removal and how it is transformed into chips. Also, additional measurements can be made, e.g., of chip size or chip formation angle. The same is not feasible with milling processes. In milling, the rotary motion of the tool causes the point at which the material is removed to constantly change, allowing only a general observation of this phenomenon. This also implies fewer possibilities for further analyses. Despite this, the need to conduct research in this area is justified. This is especially true in the field of precision milling, where the minimum undeformed chip thickness is a key aspect of this machining method. In addition to that, the number of available studies addressing this problem in relation to magnesium alloys is negligible. Especially when compared to other materials for which there are many publications available. Thus, the main objective of this study was to observe the cutting zone and analyze the phenomenon of material removal and chip formation during precision milling of AZ91D magnesium alloy. The study also investigated the aspect of burrs formation.

## 2. Materials and Methods

**Figure 1:**
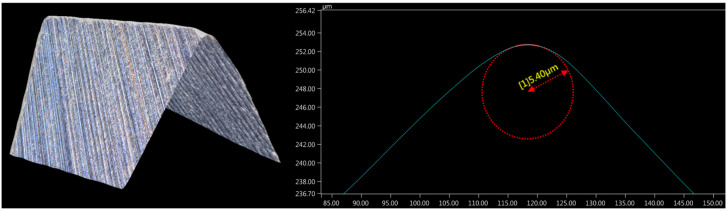
Microscopic images of a cutting edge radius.

Experiments were performed on samples of AZ91D magnesium alloy. It is the most widely used magnesium alloy. This material is lightweight and has favourable strength properties at the same time, which is why it is used in the manufacture of components for automotive, aviation, and sports equipment, among others. The samples were in the form of cuboids with the dimensions of 30 × 50 × 100 mm. The machining process was carried out on an AVIA VMC800HS milling center (Precision Machine Tools Factory AVIA S.A., Warsaw, Poland). Machining tests involved milling a slot with a width corresponding to the diameter of the cutting tool. The tests were performed using an end mill made of tungsten carbide. The end mill had no protective coating. The cutting tool had a diameter of 16 mm and two cutting flutes, and its geometry made it suitable for machining light alloys. The end mill had a helix angle of 45° and no corner radius. It should be emphasized that despite the use of an end mill with a relatively large diameter, the machining conditions used fall within the scope of precision machining. At the same time, it enables a significant increase in machining efficiency. A Keyence VHX-5000 digital microscope (Keyence, Mechelen, Belgium) was used to measure the cutting edge radius *r_n_*—Figure 1. The average value determined at ×1000 magnification was 5.4 µm. Based on this *r_n_* value, the experimental range of feed per tooth *f_z_* was determined. Since previous studies conducted on different materials have shown that the minimum undeformed chip thickness is usually lower than the cutting edge radius [48,49,50], this study was conducted with *f_z_*/*r_n_* = 0.1–1.0 in order to detect signs of material ploughing. Machining conditions are listed in Table 1.

**Figure 2:**
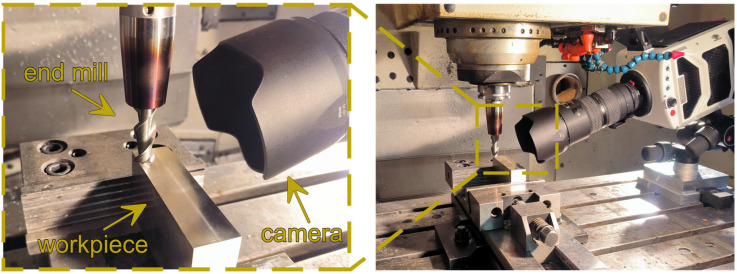
Experimental setup of precision milling.

Images were recorded using a Phantom v1610 high-speed camera (Vision Research, Wayne, NJ, USA) with a Nikkor AF 80–200 mm f/2.8D ED lens (Nikon, Tokyo, Japan). Due to a small size of formed chips and a need for more detailed observations of the material removal process, an auxiliary macro lens was used for additional magnification of the cutting zone. Captured images were further graphically corrected to improve their sharpness and quality as much as possible. The use of a high-speed camera for the study made it possible to observe phenomena occurring in the cutting zone during precision milling of magnesium alloy. Since images were recorded at 10,000 frames per second, it was possible to analyze individual stages of chip formation. A view of the cutting zone with the experimental setup and measuring equipment is shown in Figure 2. The captured images were also used to examine burrs formed during the precision milling process. The maximum width of the formed burrs was also determined using a Keyence VHX-5000 digital microscope (Keyence, Mechelen, Belgium).

In the further part of the research, measurements of the radial run-out of the cutting tool were also carried out. A Gocator 2530 laser sensor (LMI Technologies, Burnaby, BC, Canada) was used for this purpose. A laser beam was directed perpendicular to the end mill, along its axis of rotation. The tool path was determined based on a laser-captured signal and it was then used to calculate radial run-out.

## 3. Results and Discussion

**Figure 3:**
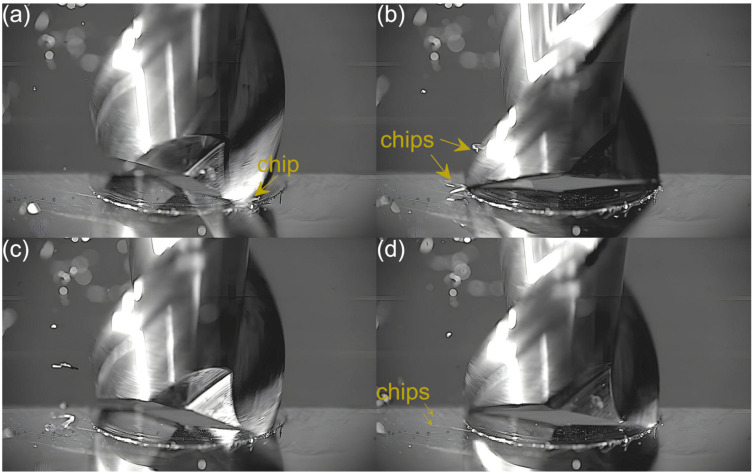
Entry and exit phases of (**a**,**b**) first flute and (**c**,**d**) second flute for *f_z_* = 0.54 µm/tooth.

**Figure 4:**
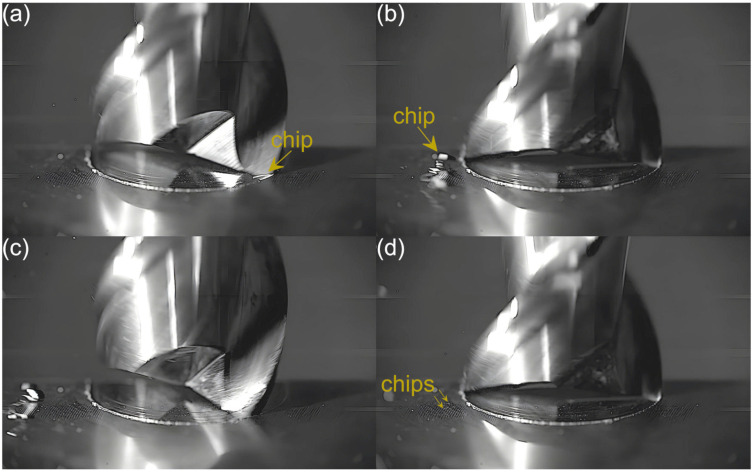
Entry and exit phases of (**a**,**b**) first flute and (**c**,**d**) second flute for *f_z_* = 1.08 µm/tooth.

**Figure 5:**
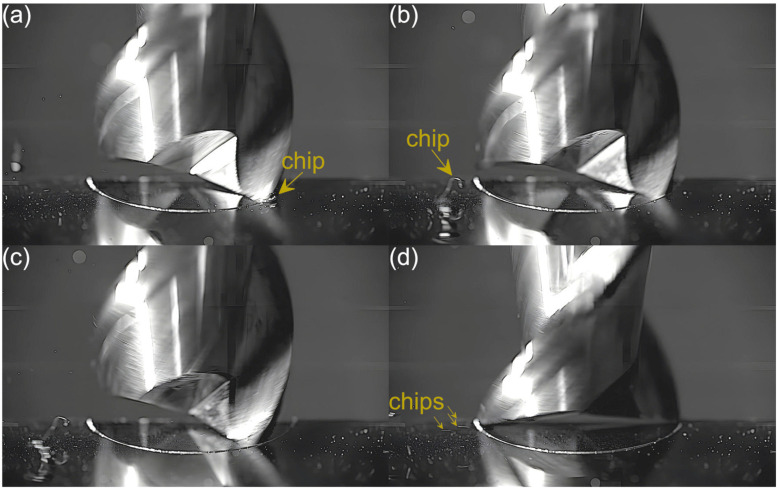
Entry and exit phases of (**a**,**b**) first flute and (**c**,**d**) second flute for *f_z_* = 1.62 µm/tooth.

**Figure 6:**
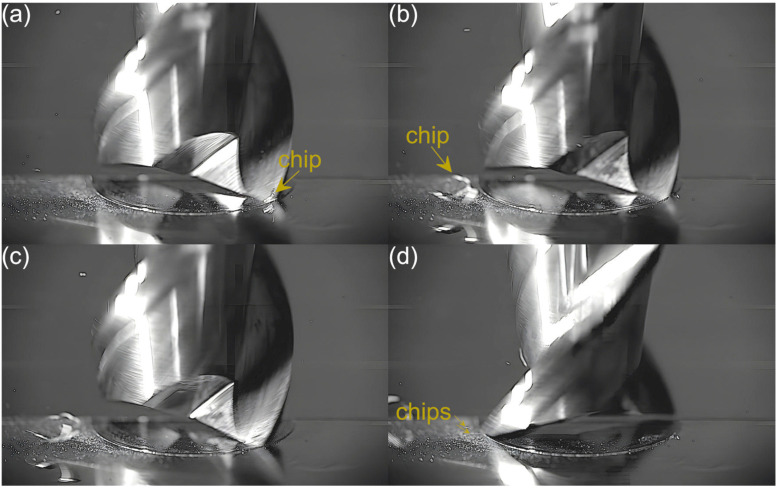
Entry and exit phases of (**a**,**b**) first flute and (**c**,**d**) second flute for *f_z_* = 2.16 µm/tooth.

**Figure 7:**
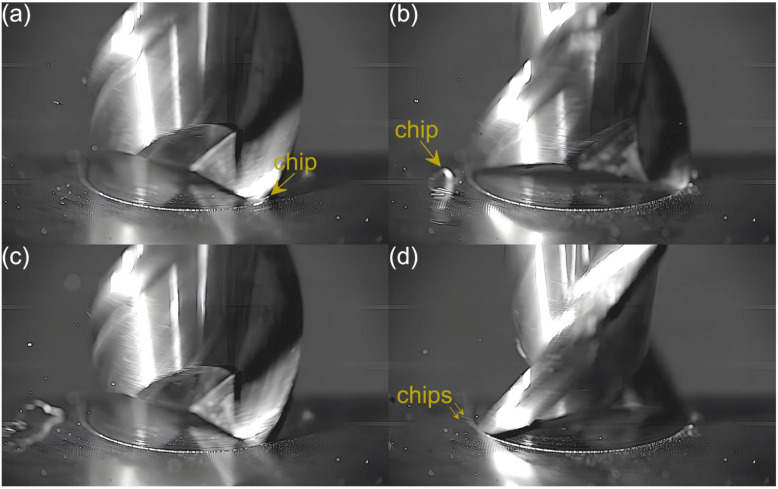
Entry and exit phases of (**a**,**b**) first flute and (**c**,**d**) second flute for *f_z_* = 2.70 µm/tooth.

**Figure 8:**
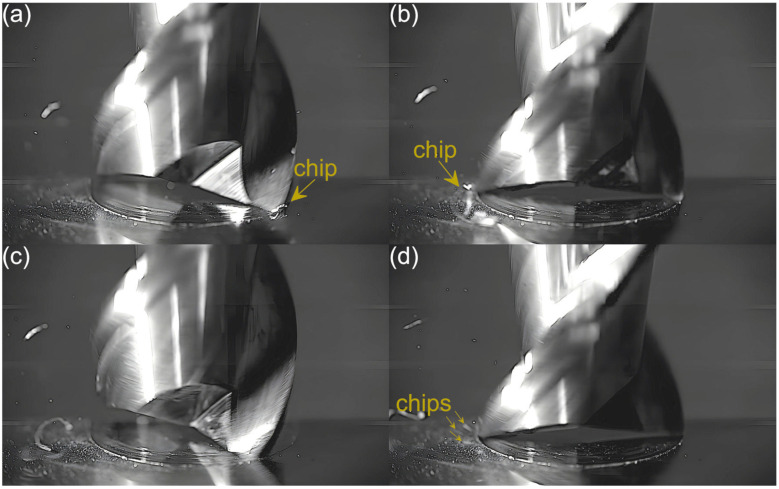
Entry and exit phases of (**a**,**b**) first flute and (**c**,**d**) second flute for *f_z_* = 3.24 µm/tooth.

**Figure 9:**
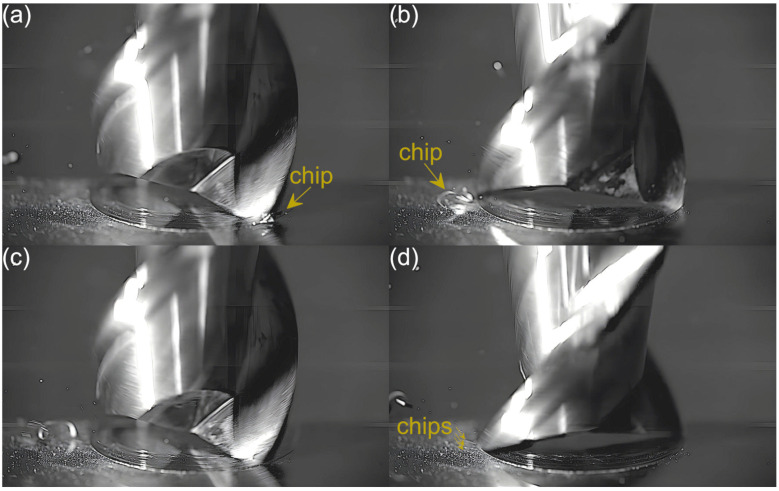
Entry and exit phases of (**a**,**b**) first flute and (**c**,**d**) second flute for *f_z_* = 3.78 µm/tooth.

**Figure 10:**
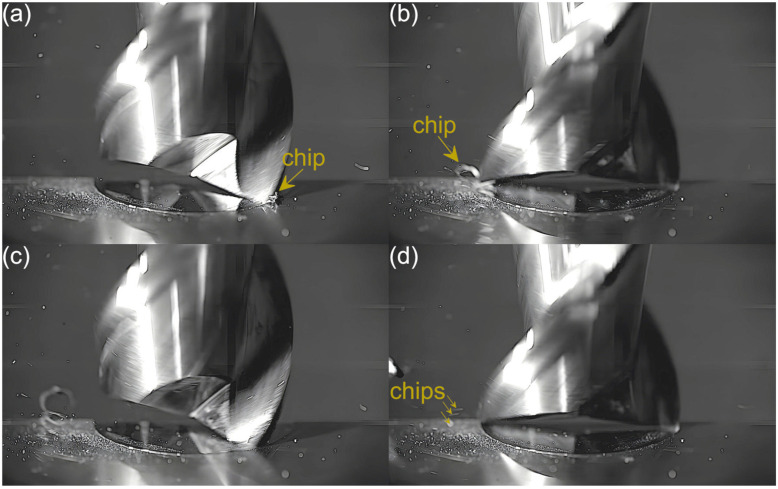
Entry and exit phases of (**a**,**b**) first flute and (**c**,**d**) second flute for *f_z_* = 4.32 µm/tooth.

**Figure 11:**
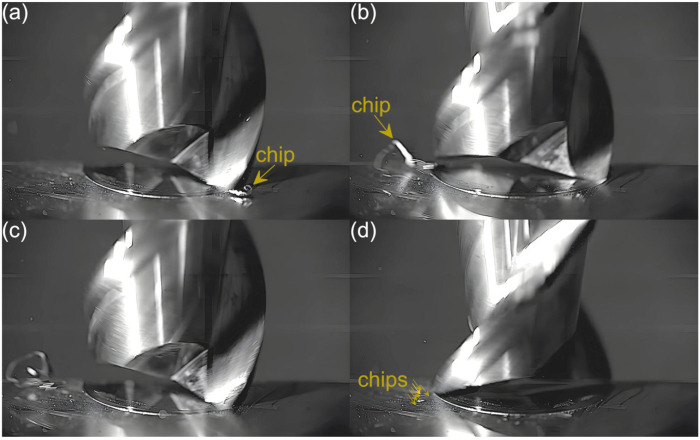
Entry and exit phases of (**a**,**b**) first flute and (**c**,**d**) second flute for *f_z_* = 4.86 µm/tooth.

**Figure 12:**
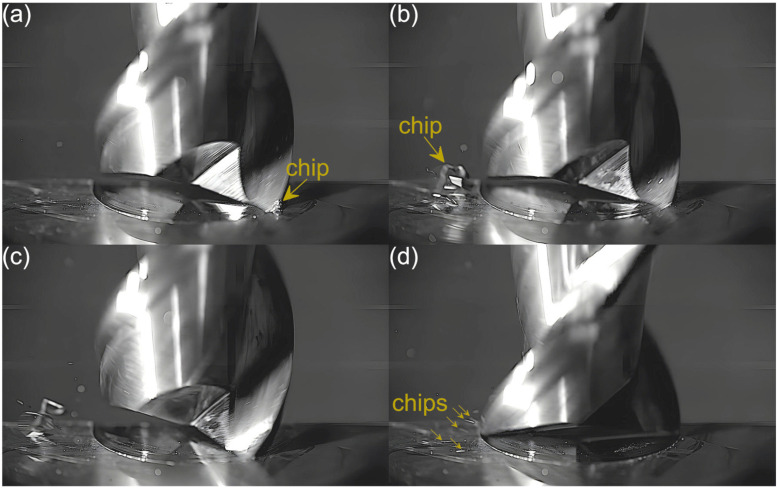
Entry and exit phases of (**a**,**b**) first flute and (**c**,**d**) second flute for *f_z_* = 5.40 µm/tooth.

Captured images were used to extract individual frames showing different stages of cutting flutes performance. Focus was put on two phases of cutting flute performance—the initial phase when chip formation begins and the final phase when the chip formation comes to the end. The analysis was carried out for both cutting flutes separately. Material removal process as a function of the feed per tooth is shown in Figure 3, Figure 4, Figure 5, Figure 6, Figure 7, Figure 8, Figure 9, Figure 10, Figure 11 and Figure 12.

Due to insufficient magnification of the high-speed camera lens, an additional lens was used to enable considerable image magnification, which was crucial for this type of research. This was necessary to capture the greatest possible close-up of the cutting area, in order to observe the material removal process at very low values of undeformed chip thicknesses. At the same time, however, the use of this additional lens made it impossible to obtain a sharp image across the entire tool width, which was due to the focal length of the lens. Although the images were processed to improve their quality, the image blurring hindered the analysis to a considerable extent.

An analysis of the machining process conducted at the lowest feed per tooth reveals that the process of chip formation by the first cutting flute begins in the initial phase of performance (Figure 3a). Although the formed chip is not clearly visible due to its small size, which results from a small amount of the removed material. Moreover, the chip is also partially obscured by the burrs formed on the edges of the slot. Once the material removal by the flute is complete, the formed chip is ejected by a centrifugal force (Figure 3b). The formed chip is relatively small. Every several rotations of the tool one can observe the presence of two chips, which may be due to either chip breakage or the other chip’s formation resulting from partial removal of the accumulated burrs. As far as the other flute is concerned, no chips are formed in the initial phase of its performance (Figure 3c). However, after completing a full movement range by the flute, one can observe the presence of very fine chips or single chip fragmentation (Figure 3d). These chips differ significantly in terms of size and shape from the chip formed by the first flute.

In the milling process conducted with a feed of 1.08 µm/tooth, the chip formed by the first flute is more visible than that formed with the lowest feed, which results from a greater undeformed chip thickness and much smaller burrs. This indicates a smaller share of plastic deformation and thus reduced ploughing. Consequently, the resulting chip is larger and continuous. As for the second flute, on the other hand, the situation resembles that observed for the lowest feed. No chips are formed in the entry phase of tool performance, while only very fine chips or chip fragments are visible in the exit phase.

Despite further increases in feed per tooth, the course of the material removal process remains similar. The chip formed by the first flute is large and clearly visible, even in the initial phase of tool performance. The second flute, on the other hand, forms fine chips that are not visible until the flute performance is completed. The main difference with respect to lower feeds is a gradual increase in the size of the chip formed by the first flute. One can also observe an increase in the number and size of fine chips that are visible during the performance of the other flute. This is due to an increasing undeformed chip thickness of material.

One of the common causes of differences in the size of formed chips is the material ploughing phenomenon [51,52]. If the undeformed chip thickness is too small for the flute to initiate the shearing process, the material is not removed. Only after sufficient material has accumulated it is removed by another flute. In the case of the conducted research, it seems that the differences in chip size are too large to be caused by material ploughing. The size of the chips does not usually differ that much, and a chip was formed with each rotation of the cutting tool. Moreover, the change was small even at higher feed values. A more probable cause of the difference in the size of chips formed by both cutting flutes seems to be the radial run-out of the tool [53,54,55]. The uneven rotation of the end mill would cause one flute to be more protruded, which would result in greater material removal than for the other flute, which would be retracted.

**Figure 13:**
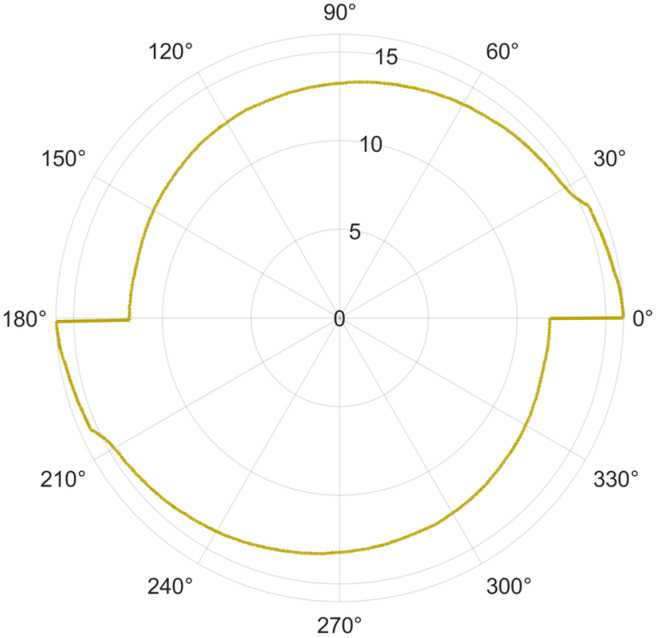
Trajectory of end mill.

To determine the cause of the non-uniform material removal by the cutting flutes, the tool’s radial run-out was additionally measured, appearing to be a potential cause of this phenomenon. An example of cutting tool trajectory is shown in Figure 13. The average value was 3.0 µm. Thus, the measurements confirm the occurrence of radial run-out of the tool. However, this value seems insufficient to cause such large differences in the size of chips formed by the both cutting flutes. This is because the radial run-out is lower than the highest applied feed per tooth at which only fine chips were formed by the other flute. The obtained radial run-out value does not therefore explain the observed phenomenon, and its cause should be sought in other structural or dynamic conditions affecting the performance characteristics of the system.

**Figure 14:**
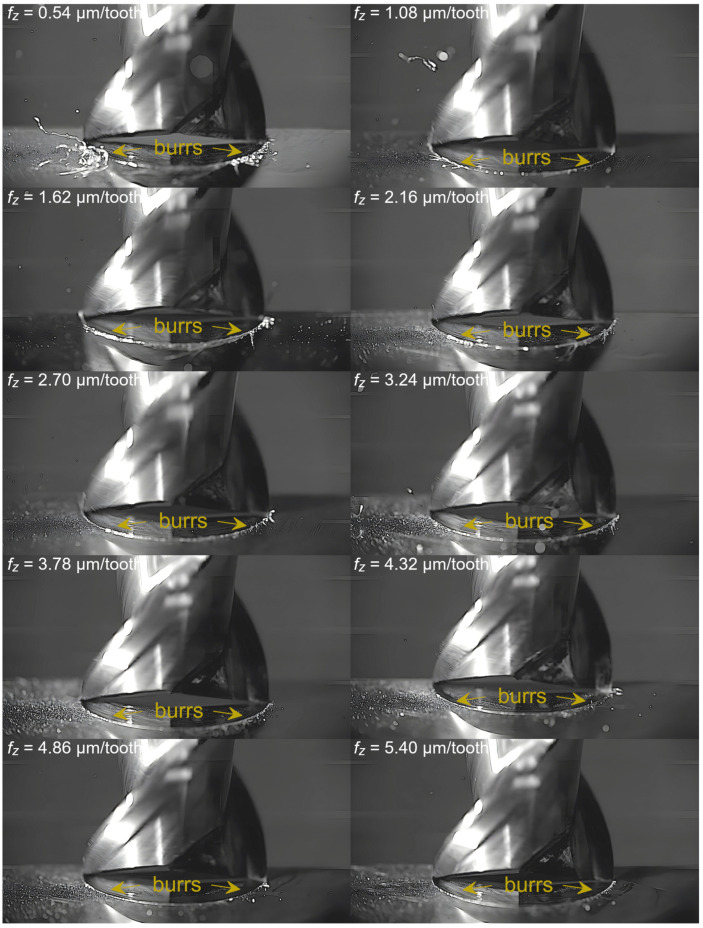
Burrs for different *f_z_* values.

**Figure 15:**
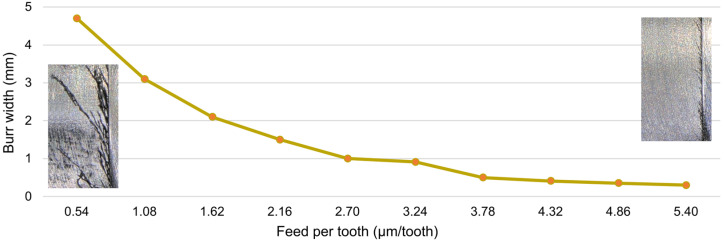
Maximum burr width for different *f_z_* values.

The formation of burrs on the edge of the milled slot is another important problem observed by image recording. Figure 14 presents the largest burrs formed during machining conducted with different feeds. The burrs formed along the entire length of the slot were also measured and their maximum width was determined—Figure 15. The formation of burrs is most evident when the lowest feed values are used. This is related to material ploughing resulting from the low undeformed chip thickness. When the amount of material is insufficient, the flute cannot initiate the shearing process. As a result, the material undergoes plastic deformation, which promotes the formation of burrs. Large burrs have a negative impact on the machining process and the workpiece itself. First of all, they cause deterioration of surface quality. Moreover, additional operations are required to remove them, which makes the entire machining process longer and can negatively affect manufacturing accuracy. A large number of burrs also causes the newly formed chips to become entangled in them, leading to their accumulation on the edge of the slot. This phenomenon is also undesired. Chip clusters can prevent material removal and, more importantly, cause an increase in the temperature generated in the cutting zone, which is a key aspect when machining magnesium alloys.

As the feed is increased, the number and size of burrs decrease. This indicates reduced susceptibility of the material to plastic deformation and, at the same time, the receding of ploughing, which is due to a higher and higher undeformed chip thickness. An increase in the feed to 1.08 µm/tooth results in a significant reduction in burrs, as a result of which the chips stop accumulating against the slot edge. Large burrs are not formed over the entire outline of the slot, but only in its side sectors, i.e., where the undeformed chip thickness is the lowest. Small burrs are only visible in the central zone of flute performance, where the undeformed chip thickness holds maximum values. This confirms that the occurrence of plastic deformation promotes the formation of burrs. From a feed of 2.70 µm/tooth, burrs mainly occur in the entry zone of the flute, which can be related to hindered initiation of the shearing process by the cutting flute. This is because the undeformed chip thickness in this zone starts at zero and gradually increases. The too small amount of material prevents the initiation of material shearing. This problem does not occur on the opposite side of the slot, where the undeformed chip thickness changes from maximum to zero. In the milling process conducted with a feed of 3.78 µm/tooth, mainly fine burrs are visible, with only single larger burr occurring locally. They still occur mainly in the entry zone of the cutting flute. At the highest feed, on the other hand, only fine burrs can be observed. The undeformed chip thickness for this feed value is therefore sufficient to minimize the impact of plastic deformation and the tendency for burrs to occur. Measurements of chip width confirm the relationships observed at the front of the slot. The greatest burr widths occurred at the lowest values of feed per tooth, after which they gradually decreased. The greatest changes in width occurred at the lower feed range. As the feed value increased, the changes became smaller, especially from approx. 3.78 µm/tooth.

## 4. Conclusions

This study focused on the problem of material removal and chip formation process during precision milling of AZ91D magnesium alloy. The following conclusions can be drawn from the observations of the cutting zone:

(1) Both the cutting flutes of the end mill did not perform at a uniform rate, as the material was mainly removed by the first flute. In effect, the size of chips formed by the flutes differed to a relatively great extent. Although the study confirmed the presence of a small radial run-out of the end mill, its value seems too low to fully explain the non-uniform performance of the two cutting flutes. The differences are so large that the ploughing phenomenon does not seem to explain them either.
(2) The chip size increased with feed per tooth, which resulted from the removal of increasing undeformed chip thickness of material. However, this is mainly visible for chips formed by the first flute, because chips formed by the second flute are very fine despite the increase in feed.
(3) The use of low feed per tooth in the machining process led to the formation of very big burrs, which indicates the presence of intensive plastic deformation and material ploughing. However, already at a feed per tooth of approx. 2.7 µm/tooth there was a significant reduction in burrs, so it can be assumed that this is a transition zone between the ploughing dominant zone and shearing dominant zone.
(4) The shearing process by the first cutting flute initiated at a feed per tooth of 0.54 µm/tooth and thus at a ratio of *f_z_*/*r_n_* = 0.1. Nevertheless, given the non-uniform performance of the cutting flutes, this value cannot be taken to be the minimum undeformed chip thickness.

The results provided valuable insight into the mechanism of material removal during precision machining of AZ91D magnesium alloy. In particular, the results of this study confirmed that the shearing process of this material could be conducted with the feed values lower than the cutting edge radius. However, additional research is necessary to clarify the exact cause of non-uniform chip formation. The potential main causes, i.e., tool radial run-out and material ploughing, do not seem to fully explain this phenomenon. In this way, it will be possible to determine the minimum undeformed chip thickness. In future studies, the research problem will be extended to include other magnesium alloys and other cutting tools. A more detailed analysis of the chips themselves is also planned, as well as other machinability indicators.

## Figures and Tables

**Table 1:** Machining conditions.

Test No.	Feed per Tooth (µm/tooth)	Cutting Speed (m/min)	Axial Depth of Cut (mm)	Radial Depth of Cut (mm)
1	0.54	800	0.15	16
2	1.08	800	0.15	16
3	1.62	800	0.15	16
4	2.16	800	0.15	16
5	2.70	800	0.15	16
6	3.24	800	0.15	16
7	3.78	800	0.15	16
8	4.32	800	0.15	16
9	4.86	800	0.15	16
10	5.40	800	0.15	16

## Data Availability

The original data presented in the study are openly available in Zenodo repository at https://doi.org/10.5281/zenodo.17594029.
